# Human Cytomegalovirus Congenital (cCMV) Infection Following Primary and Nonprimary Maternal Infection: Perspectives of Prevention through Vaccine Development

**DOI:** 10.3390/vaccines8020194

**Published:** 2020-04-23

**Authors:** Giuseppe Gerna, Daniele Lilleri

**Affiliations:** Laboratories of Genetics, Transplantology and Cardiovascular Diseases, Fondazione IRCCS Policlinico San Matteo, 27100 Pavia, Italy; d.lilleri@smatteo.pv.it

**Keywords:** congenital cytomegalovirus, primary infection, non-primary infection, gB, neutralizing antibodies, pentameric complex, bacterial artificial chromosome, dense bodies, cytotrophoblast, hyperimmune globulin

## Abstract

Congenital cytomegalovirus (cCMV) might occur as a result of the human cytomegalovirus (HCMV) primary (PI) or nonprimary infection (NPI) in pregnant women. Immune correlates of protection against cCMV have been partly identified only for PI. Following either PI or NPI, HCMV strains undergo latency. From a diagnostic standpoint, while the serological criteria for the diagnosis of PI are well-established, those for the diagnosis of NPI are still incomplete. Thus far, a recombinant gB subunit vaccine has provided the best results in terms of partial protection. This partial efficacy was hypothetically attributed to the post-fusion instead of the pre-fusion conformation of the gB present in the vaccine. Future efforts should be addressed to verify whether a new recombinant gB pre-fusion vaccine would provide better results in terms of prevention of both PI and NPI. It is still a matter of debate whether human hyperimmune globulin are able to protect from HCMV vertical transmission. In conclusion, the development of an HCMV vaccine that would prevent a significant portion of PI would be a major step forward in the development of a vaccine for both PI and NPI.

## 1. Introduction

In the last few years, congenital human cytomegalovirus (cCMV) infection has been identified to be a result of both maternal primary and nonprimary infections. This new pathogenetic approach has created a series of new problems both in the differential diagnosis and vaccine prevention of cCMV, following the two types of maternal infection. In this review, we report the epidemiological immunological and clinical features of both types of maternal infections, then we examine the molecular and virological methods to discriminate between the two types of infection, and finally we analyze the approaches of passive and active immunoprophylaxis for the prevention of cCMV. Last, some local pathological and pathogenetic aspects of cCMV at the placental–uterine interface are reported.

## 2. Primary Infections

(a) *Epidemiology of congenital cytomegalovirus (cCMV) infections following maternal primary infection (PI).* In Western Europe, North America and Australia, human cytomegalovirus (HCMV) PI occurs in childhood and adulthood in 50–70% of women in the fertility age range, thus, a high percentage (30–50%) of women in childbearing ages might undergo PI. It is generally believed that the transmission rate to the fetus in pregnancy during PI of seronegative women might occur in 30–40% of cases [1]. Recent studies have reported a similar trend [2,3,4]. 

(b) *Immune response.* Both innate and adaptive (antibody and T-cell) immune responses are involved in the control of the HCMV infection. Within the innate response, the protective role of: (i) NK cells and, particularly, CD57^+^ NKG2C^bright^ cells [5], (ii) antibody-dependent (AD) cellular mechanisms [6,7], and (iii) γ/δ T-cells and, particularly, Vδ2^-^ γ/δ T-cells [8,9], against HCMV transmission to the fetus, has not been fully investigated. Thus, at the moment, there are no innate immune correlates that distinguish between transmitting (T) and non-transmitting (NT) women [9].

Both neutralizing antibodies (NAb) and non-neutralizing binding antibodies have been believed to exert a protective effect against HCMV transmission to the fetus in seronegative pregnant women [10,11]. However, a more recent randomized study did not confirm the protective effect of commercial human immunoglobulin (HIG) preparations in the prevention of cCMV infections, in comparison to non-treated controls [2]. At the moment, the protective role of HIG in the prevention of cCMV awaits confirmation from more extended controlled studies. 

Up until 2004, three HCMV glycoprotein complexes (gCs) were known to be the major targets of the NAb response—(i) gB (gCI), homotrimer coded by UL55; (ii) gM/gN complex (gCII), consisting of UL100-coded gM and UL73-coded gN; and (iii) gH/gL/gO complex (gCIII), consisting of UL75-coded gH, UL115-coded gL, and UL74-coded gO. In 2004–2005 the UL128-130-131 locus (referred to as UL128L) was found to be indispensable for infections of endothelial [12] and epithelial [13] cells. Subsequently, UL128L gene products were found to be complexed with gH/gL to form the pentameter complex (PC) gH/gL/pUL128L [14]. Then in 2010, a series of potently neutralizing human monoclonal antibodies (mAbs) were isolated from the blood of seropositive subjects [15]. These mAbs—(i) were reactive with the gene products of the UL128L; (ii) neutralized infections of the epithelial and endothelial cells, but not fibroblasts, in the picomolar range, unlike mAbs to gB, gH, gM/gN, which neutralized the infection of all target cells tested in the nanomolar range; and (iii) were also elicited following the immunization of mice with PC [16]. 

Using these mAbs reactivity with 10 different PC epitopes, and a competitive ELISA assay [referred to as inhibition of the mAb binding (IMAB) assay] in which the PC bound to the solid-phase was competitively reacted with human sera and murinized human mAbs, it was found that in pregnant women with PI, IgG antibodies to some PC epitopes appear earlier in NT compared to the T mothers [17]. Subsequently, the IMAB titers were shown to be significantly higher for 7/10 PC antigenic sites in NT compared to T mothers [18]. Furthermore, the number of antigenic sites recognized by T women was found to be significantly lower than the number of sites recognized by NT women during the first and the second month after onset of infection, whereas no difference was detected from the third month onward. Thus, human mAbs provided with a potent neutralizing activity and directed to multiple epitopes of UL128L gene products, might represent important immune correlates of protection against vertical HCMV transmission.

Finally, the blocking activity of human antibodies in preventing or limiting virus dissemination was investigated in T and NT pregnant women by evaluating the relevant serum inhibition of—(i) viral plaque formation (PFI) or cell-to-cell spreading of the virus; (ii) virus leukocyte transfer (LTI) from infected endothelial cells, with virus dissemination through the blood stream; and (iii) viral syncytium formation (SFI), an important alternative route of virus dissemination instead of cell-free virus infection. While LTI and SFI antibodies were found to appear at least 30 days after onset of PI in pregnant women, PFI antibodies were shown to appear much earlier and to reach significantly higher titers in NT women during the first and the second month, after the onset of PI compared to the T women [18]. As for human mAbs, those directed against the PC displayed the highest PFI activity, unlike the mAbs directed to gB and gH [18].

**Figure 1:**
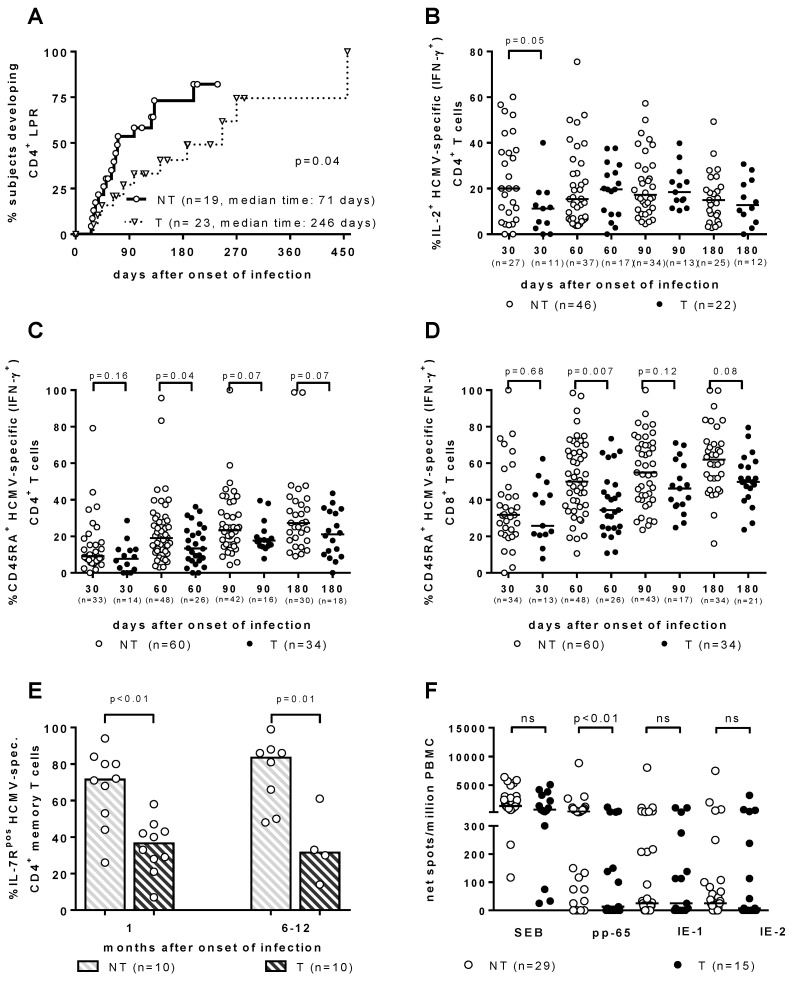
Immune correlates of protection against human cytomegalovirus (HCMV) vertical transmission. (**A**) CD4^+^ LPR occurs significantly earlier in NT vs. T women. [20]. (**B**) IL-2^+^ HCMV-specific (IFN-γ^+^) CD4^+^ T cells are significantly higher in NT vs. T pregnant women in the first month after infection onset [22]. (**C**) CD4^+^ and (**D**) CD8^+^ CD45RA^+^ HCMV-specific (IFN-γ^+)^ T cells are significantly higher in NT vs. T pregnant women in the second month after infection onset [22]. (**E**) IL-7R^pos^ HCMV-specific CD4^+^ memory T cells are significantly higher in NT vs. T pregnant women both at 1 and 6–12 months after infection onset [23]. (**F**) pp65-specific spots/million peripheral blood mononuclear cells are significantly higher in NT vs. T pregnant women, while no difference in response to IE-1 or IE-2 was observed [24].

As for the T-cell response, it has been traditionally considered to be the major barrier against HCMV vertical transmission. However, throughout the years, the search for the immune correlates of HCMV transmission to the fetus in T and NT pregnant women with PI has provided some interesting results, which are all relevant to a significant delay in the development of a specific immunological function. (Figure 1). First, the HCMV lymphoproliferative response occurred significantly later in T vs. NT women [19,20] (Figure 1A). Second, the re-expression of CD45RA^+^ T_EMRA_ cells was significantly delayed in the T women, within both HCMV-specific IFN-γ^+^ CD4^+^ and CD8^+^ T-cell subsets [21] (Figure 1C,D). Third, HCMV-specific CD4^+^ T-cells showed a significant delay in IL-2 production in T vs. NT pregnant women [22] (Figure 1B). Fourth, IL-7R^pos^ long-term memory HCMV-specific CD4^+^ T-cells, when developed significantly later in T vs. NT women, were associated with HCMV vertical transmission [23] (Figure 1E). Finally, in recent years a cultured ELISPOT assay based on a 2-week double peripheral blood mononuclear cell stimulation with peptide pools of HCMV proteins, has shown a lymphoproliferative response to pp65 (but not to IE-1 or IE-2), which was significantly higher in NT vs. T mothers [24] (Figure 1F). In addition, in a multivariate analysis, the cultured ELISPOT response, as well as a higher avidity index and a lower viral load, were found to be factors independently associated with a lower risk of cCMV [24]. 

(c) *Clinical outcome*. cCMV infection following HCMV PI at birth might be symptomatic (10%) or asymptomatic (90% of the cases). Among symptomatic cases, about 5% present typical symptoms of cytomegalic inclusion disease (CID)—jaundice, purpura, hepatosplenomegaly, thrombocytopenia, microcephaly, intracranial calcifications, while the remaining 5% show milder symptoms [25]. The most common sequelae of cCMV are sensorineural hearing loss (SNHL), which is more frequent in symptomatic infections [26], as well as permanent intellectual and physical disabilities [27].

## 3. HCMV Re-Infection or Nonprimary Infection

*Epidemiology of cCMV following maternal non-primary infection (NPI)*. While in the initial years of laboratory and clinical management of HCMV infections it was generally believed that cCMV infections were the result of HCMV PI in pregnant seronegative women, in the last two decades of the 20th century several reports have described cases of cCMV in seropositive women. While in reports from the US cases of cCMV occurring in seropositive mothers were described as asymptomatic or subclinical (unlike symptomatic cCMV cases which were considered to be the result of a PI [10,28,29]), reports from other countries, such as England [30], Belgium [31], and Sweden [32,33] indicated that HCMV maternal NPI could be a major cause of the cCMV disease. Subsequently, US researchers observed similar findings, i.e., that NPI could be the cause of cCMV [34,35]. Importantly, in a long-term study on 16,474 newborns from Sweden, it was reported that of 76 congenitally infected infants, 22/76 (29%) showed transitory neonatal symptoms, while 11/60 (18%) were affected by neurological symptoms by the age of 7 years, as a result of either HCMV PI or NPI [33]. In the meantime, it was reported that NPI could be the result of either the reactivation of a latent virus [28] or the re-infection by a new virus strain [35]. These findings led to the conclusion that NPI could cause a number of cCMVs as high as PI with a comparable number of CID cases.

A meta-analysis study of the epidemiology of cCMV infection reported an overall birth prevalence of 0.64%, based on a list of 55 articles found in the MEDLINE database for the period of 1966–2006, with 11% symptomatic live-born infants [1]. In the same study, the combined birth/fetal prevalence of cCMV infection in pregnant women with PI was 32.3% (29.8–34.9%) in 19 study groups, whereas it was 1.4% (1.1–1.7%) in pregnant women with NPI. The reason for this striking difference between PI and NPI in the rate of fetal HCMV transmission in pregnant women was likely to reside in the partial protection against new HCMV infections, as conferred by the pre-conceptional immunity, which was mediated by close contacts within households, in association with socioeconomic factors [36,37]. However, other studies have reported a contribution to cCMV of NPI in countries with high HCMV seroprevalence. This contribution appeared to be similar or even superior to that of PI in highly seroimmune maternal populations [38,39] and even in some groups of healthy seroimmune women in the US [40]. 

These epidemiological findings implied that the immune correlates of protection reported above for PI might only partially protect seropositive pregnant women from reinfection. From this conclusion a series of concerns aimed at the development of an HCMV vaccine arises, which should be directed to protection not only against PI, but also against NPI. Thus, new immune correlates of protection against NPI should be investigated and hopefully identified. In the meantime, NPI were attributed to a re-infection caused by a strain different from the one causing PI [35] and were partially diagnosed by an ELISA assay detecting antibodies to ≥1 of four polymorphic epitopes of the two fragments each of gH and gB of the two laboratory strains AD169 and Towne. These fragments were found to be reactive with strain-specific antibodies in 60% of the subjects tested [41] and appeared to be provided with a protective role in strain-specific immunity [40].

*Immune response.* Apart from the above-mentioned study aimed at identifying a strain-specific antibody response elicited during an NPI by a new virus strain, no other studies for either the serological identification of HCMV reinfections or for the investigation of immune correlates of protection against NPI have thus far been reported. However, by taking advantage of the methodological approach of Novak et al. [41], several epidemiological investigations were conducted, showing that the rate of cCMV infections following NPI might reach and even overcome that of cCMV, following PI [38,40,42]. Very recently, it was reported that NAb to the trimer complex (TC) gH/gL/gO reached the same titer as NAb to the PC during NPI of both seropositive transplant recipients and pregnant women [43]. This result prompted the authors to conclude that these two groups of NAb do not display any protective effect against HCMV transmission to the fetus during NPI, since they were detected at the same titer in both T and NT pregnant women. However, these conclusions must be taken with caution, since (i) protection from fetal transmission by NAb to PC was reported in pregnancy only for the first two months after PI onset, which then disappeared [18]; (ii) protection against transmission by neutralizing NAb to either PC or TC remained to be investigated in HCMV-seropositive pregnant women, in whom the kinetics of the antibody response to either PC or TC must be totally different compared to PI; (iii) some mechanisms other than neutralization, such as antibody-dependent (AD) cellular immunity, must still be investigated, as well as T-cell adaptive immunity.

*Clinical outcome.* Due to the documented direct relationship between high seroimmune prevalence and the rate of cCMV [44], most population studies were based on the documented (or presumed) correlation between pre-pregnant HCMV seropositivity and the rate of cCMV. On this basis, while the rate of cCMV in Europe and North America ranged from 0.18% to 0.48%, in developing countries, such as Brazil, and several African and Asian countries, it ranged from 1% to 2% [45], i.e., it was much higher. In addition, while for a long time pre-existing maternal immunity was associated with less severe cCMV and a lower rate of severe hearing loss [39], other studies have reported that the frequency of hearing loss and neurodevelopmental sequelae was comparable in children born to mothers with PI or NPI [38,39,40,46].

## 4. HCMV Latency 

**Figure 2:**
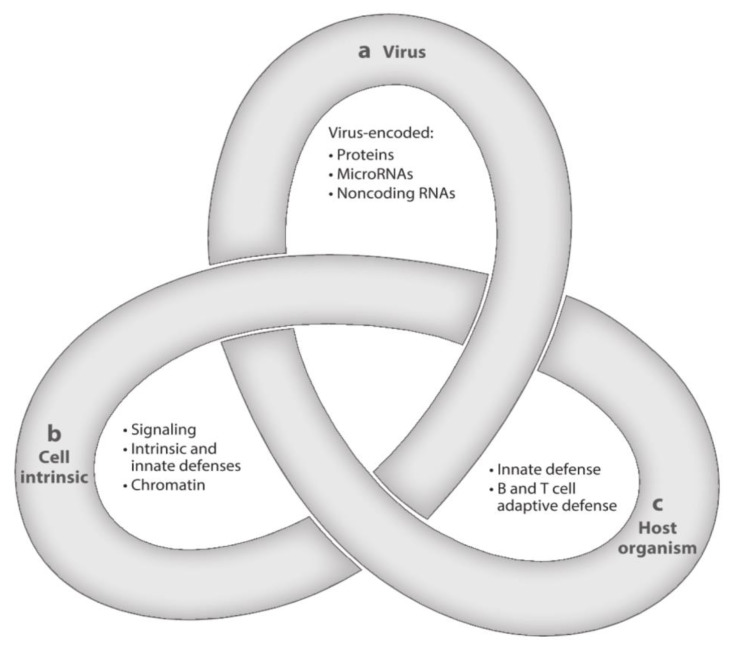
Latency is a complex multifactorial process resembling a Gordian Knot. It is regulated by (**a**) viral factors, (**b**) cellular factors (cellular signaling and chromatin remodeling), and (**c**) innate and adaptive host immune responses. These three aspects of HCMV latency/persistence include latent, chronic, and replicative states of infection, which are closely related to one another and still await to be fully clarified ([47], with permission).

After resolution of PI, HCMV, the prototype of betaherpesviruses, does not disappear from the body, but initiates a lifelong persistence, including states of latent, chronic, and replicative infection. HCMV is strictly species-specific, but displays a broad tropism for different cell types. The often recurring transition from latency to chronic or replicative infection is a complex process that is regulated by three groups of factors—viral, cell-type specific, and related to both the innate and the adaptive host immune responses. The complexity of these interactions has been assimilated to a Gordian knot (Figure 2), in that it is very difficult to explore and untangle [47]. The transition from the latent to the chronic or replicative state might occur even in healthy people, but does occur much more extensively in immunocompromised (AIDS, transplanted) patients, due to episodes of transitory or prolonged immunodeficiency, often resulting in life-threatening clinical syndromes. 

Following PI, human cells thus far identified as primary sites of latency are CD34^+^ hemopoietic progenitor cells (HPC) and CD14^+^ monocytes [48,49,50], which were found to harbor the HCMV genome in the absence of virus replication, thus representing the first cells identified as latent virus reservoirs. Latent HCMV genome has been shown to be present in a few cells at a very low copy number (about 1 genome/10^4^–10^5^ mononuclear cells), as observed in G-CSF-stimulated donors [51]. However, the HCMV latency issue has not been extensively investigated in the search for other latent reservoirs, and it is plausible that other reservoirs, as well as different latency programs might exist, in light of the different cell populations infected, as already observed for Epstein–Barr virus (EBV) latency [52].

Due to the strict species-specificity of HCMV, most latency studies have been conducted in in vitro models. The most successful model was represented by the HPC coculture with stromal cells, which was able to support HCMV latency [53,54,55,56]. This model was used for the comparative study of the states of HCMV latency and reactivation of different virus strains in HPC subpopulations. Using a GFP fluorescent viral protein as a marker for the selection of a pure population of HPC infected cells, it was possible to distinguish HPC expressing GFP (and therefore containing the viral genome) from HPCs not expressing GFP (and therefore not infected and not containing the viral genome) [53]. On the other hand, humanized mice have thus far represented the only in vivo model for the study of the HCMV latency and reactivation in the presence of a functional immune system [57].

*Viral genetic determinants of latency.* While the subsequent activation of immediate–early (IE), early, and late genes has been well-defined during the productive HCMV infection of human fibroblasts, viral gene expression in other cell types and, particularly, in cells undergoing viral latency, is still somewhat controversial. However, both in vitro studies as well as latently infected mice have shown expression of IE and early genes, in the absence of a virus progeny [54,58,59].

Following repeated passages in cultured fibroblasts, wild-type HCMV strains were found to undergo a series of genomic rearrangements, among which the most extended was the loss of the ULb’ region [60]. This region consists of approximately 15 kb encompassing UL132-150, whose functions were important for cell tropism, immune evasion, and virus latency. In particular, the UL133-138 gene locus included the four genes UL133, UL135, UL136, and UL138, whose product functions had to be considered altogether within the entire locus. In greater detail, the synergistic and antagonistic interactions between the different proteins encoded by this locus seemed to regulate the latency. It was suggested that the antagonistic function of UL135 (replication activating) and UL138 (replication suppressive) make the proteins encoded by the locus a sort of molecular switch regulating the states of latency and replication [47]. In addition, within the UL133-UL138 locus, UL136 has been reported to express five protein isoforms (UL136-33kDa, UL136-26kDa, UL136-25kDa, UL136-23kDa, and UL136-19kDa), which are dispensable for virus replication in human fibroblasts, but have both antagonistic and synergistic functions in endothelial cells, HPC, and humanized mice [61]. UL136 has been considered to be a smaller locus within the broader UL133-138 locus, with UL136-33kDa and UL136-26kDa isoforms promoting replication, and UL136-23kDa and UL136-19kDa isoforms antagonizing replication, while UL136-25kDa would promote latency in CD34^+^ HPC, but replication in humanized mice, thus, acting as a balance between replication (UL135-dominant) and latency (UL138-dominant) [47].

In conclusion, HCMV latency is a complex multifactorial process, as already mentioned. The integrated analysis of multiple factors (most of which are still unknown) will permit a satisfactory understanding of the molecular pathogenesis of the state of latency, as well as the definition of whether or not only cells of the hematological lineage are permissive for latency.

## 5. Sequencing of the Viral Genome

More than sixty years after the discovery of HCMV, the genetic integrity of HCMV was largely compromised by both the large series of virus passages in cell cultures and the high levels of genetic variability among HCMV genomes. The first HCMV strains were reported by: (i) Margaret G. Smith from two babies with CID [62], (ii) Thomas H. Weller, who isolated the Davis strain from a liver biopsy, as well as the Kerr and Esp strains from neonates with CID [63]; and (iii) Wallace P. Rowe, who recovered AD169 from the adenoid tissue of a young girl [64], and also documented that the three strain groups were related to each other, largely diffused in the human population and often associated with CID. In the following decades, molecular virology studies were performed using: (i) the AD169 strain [64], and: (ii) the Towne strain, which was developed as an attenuated HCMV vaccine, following 125 passages in cell cultures [65]. 

Forty years after the discovery of HCMV, Cha et al. [60]. characterized the HCMV genomes of both laboratory strains AD169 and Towne as well as clinical isolates, showing that both AD169 and Towne were affected (compared to clinical isolates) by a large deletion of 15 and 13 kb, respectively. This deletion was located at the right end of the unique long region (UL) and was named ULb’. By comparing the AD169 genome sequences of three AD169 variants obtained from three different laboratories, it was found that all three variants had a total or partial deletion of ULb’, along with multiple genetic mutations, which were different for the three variants. Similar results were observed with Towne [66]. These findings generated the shared conclusion that any HCMV strain propagated in cell cultures was subject to harboring genetic mutations, compared to the relevant clinical strains present in clinical samples collected for virus isolation. 

In 2010, an informative study was conducted by infecting fibroblasts, epithelial and endothelial cells with three low-passaged HCMV strains for 50–63 passages, prior to sequencing the relevant genomes at different passages and comparing sequences with the original clinical samples [67]. Mutations were observed in all viruses, first in the gene RL13 (passages 8–16), then in UL128L (passages 15–20), and eventually sometimes in ULb’ in the region UL140-145 (at passages 32–63). The general conclusion drawn from this study was that all HCMV isolates were genetically unstable in all cell types and, as a consequence, all genome sequences of the passaged HCMV isolates had to be compared with those of the original clinical samples to detect the mutations that occurred during propagation in cell cultures. To obviate genetic mutations intervening in virus isolates during cell culture propagation, it was decided to clone HCMV as an infectious bacterial artificial chromosome (BAC) [67], using a methodological approach already adopted by German researchers [68,69]. BAC cloning was used for sequencing of both laboratory strains AD169 [69,70] and Towne [71,72], as well as wild-type isolates (following a variable number of passages in cell cultures) such as Toledo, PH, and TR [73], FIX (VR1814) [74], and TB40/E [75].

However, all these BAC genomes stably contained the vector cassette, which replaced the genes US2, US3, US6, and sometimes US11, thus, interfering with the regulation of MHC-I and MHC-II molecules and the T-cell and NK cell immune responses [76]. In addition, none of these BAC-cloned HCMV strains could be compared with the original virus, due to the unavailability of the original clinical samples. As a consequence, it was decided to insert the entire genome of the HCMV strain Merlin, into a BAC plasmid at passage 5, while the complete genome sequence was obtained at passage 3 [77]. This genome sequence became the first WHO International HCMV Standard [78]. However, in order to make a clean BAC-derived complete genome available from Merlin, the vector cassette was made self-excisable by using the Cre/LoxP recombination [79]. Thus, the BAC-cloned Merlin genome differed from the parental genome for the presence of a single nucleotide substitution in UL 128, and a variety of mutations in RL13, which were thought to have been acquired prior to BAC cloning and then repaired to match the original sequence. Following repair, the BAC-derived Merlin genome matched the original sequence, except for the 34-bp LoxP site after gene US28, and some minor variations concerning three non-protein-coding sequences in the b/b’ region [76]. 

As mentioned above, HCMV genomes from clinical isolates might be somewhat stabilized by BAC cloning and then reconstituted by DNA transfection. However, this type of genome integrity preservation is not absolute, and mutations might emerge not only before BAC cloning, but also during and after virus regeneration [80,81]. In order to preserve the original genome sequence and minimize the unwanted introduction of mutations, the following cautions were suggested—(i) cloning in a BAC system provided with a self-excising vector; (ii) propagating the virus in fibroblast cells; and (iii) propagating the virus in a system where RL13 and UL128L functions are conditionally repressed [81]. Repression can be removed by infecting fibroblasts not expressing the *Tet* repressor, or adding tetracycline, thus, enabling the virus to express the complete gene complement of wild-type HCMV strains. RL13 has been found to suppress virus replication in both fibroblasts and epithelial cells, while UL128L not only inhibited the virus replication in fibroblasts, but was also essential for infecting both epithelial and endothelial cells. The introduction of single-nucleotide substitutions into the UL128 of the BAC-cloned Merlin increased the cell-free virus yield and cell-to-cell spread in fibroblasts, but reduced the infection rate of epithelial cells [82]. 

## 6. Identification (Comparative Sequencing) of the HCMV Genome in PI and NPI

Genome sequencing of an HCMV strain primarily infecting a human being generally involves a single strain. Until recently, HCMV was isolated in human fibroblast cell cultures, a procedure (as reported above) introducing a series of mutations within the virus genome following a few passages. Initially, only a few hypervariable genes were sequenced by using the Sanger procedure on PCR amplicons, and the first strain to be entirely sequenced was the AD169, which was highly passaged in cell cultures and was recovered from a plasmid library [83]. Subsequently, other virus genomes from BAC preparations [75,84], extracted viral DNA [85], and PCR amplicons [67] were sequenced again using the Sanger methodology. Then, high-throughput methods were introduced [66,86,87]. Through high-throughput sequencing, an extended variability of the HCMV genome in urine samples from three neonates with cCMV infection was reported, both at the nucleotide and amino acid levels, as well as at the intra-host and inter-host levels. At this time, the HCMV genome variability was reported to be comparable to that of RNA viruses, such as HIV, because almost every HCMV ORF showed some level of intra-host diversity in the three viral populations tested [88]. Subsequently, by longitudinally testing both urine and plasma samples from congenitally infected infants, it was found that the HCMV genome might evolve in human hosts on a brief timescale, in such a way that it remains stable in the same compartment, but evolves soon after it crosses compartments of the same host, thus becoming as different as viral genomes from different hosts [89]. Viral population genetic analysis showed that both demographic events (bottleneck -decrease in the population size- and virus replication rate) and positive selection pressure might contribute to these differences.

Subsequently, the extension and patterns of HCMV genome diversity were analyzed in different clinical samples (urine, cord blood, plasma, and saliva) from congenitally infected infants [90]. This study first confirmed the high level of intra- and inter-host genomic diversity among spatiotemporally separated human hosts, and documented the distribution of genome diversity across the three different host compartments examined. However, it also showed that about 25% of the HCMV genome is conserved across patients, mostly involving genes encoding DNA-processing enzymes, capsid, tegument, and regulatory proteins. Second, the correlation of genome diversity and mutation and recombination rates was documented. Third, HCMV populations from the plasma of infants from different continents were found to be genetically similar, unlike those from urine and saliva, which were enriched in single nucleotide polymorphisms (SNPs) within glycoproteins (gB, gO, gN, and gH) and regulatory proteins, thus, suggesting the correlation of genome diversity and protein function. Finally, the intra-host diversity of clinical samples from single or mixed infections, as well the genome patterns, were overlapping.

**Figure 3:**
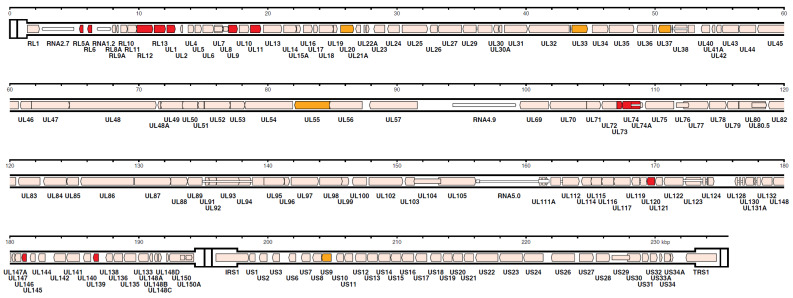
Distribution in the HCMV strain Merlin genome of the genes used for genotyping. The 12 genes (RL5A, RL6, RL12, RL13, UL1, UL9, UL11, UL73, UL74, UL120, UL146, and UL139) used for motif read-matching are in red. Two of these genes (RL13 and UL146) were also used for genotype read-matching (from Reference [92]).

However, in recent years, HCMV genome sequencing was stimulated by, both, the frequently reported introduction of mutations through repeated passages in cell cultures and the frequent finding of multiple-strain infections [86,91,92]. Using a nucleotide bait library that represented known HCMV genome diversity to select target sequences from random DNA fragments (Illumina, Agilent), Suarez et al. [92], based on the number of 244 HCMV strains sequenced by his and other groups, concluded that, when taking into account the multiple operational limitations of this methodology, the number of SNPs detected in single-strain infections was markedly reduced, compared to that of multiple-strain infections as well as to that previously reported by Renzette et al. [88]. The number of strains present in a clinical sample was estimated by two approaches—genotype read-matching and motif read-matching (Figure 3). The genotype read-matching approach included alignment of the reads to sequences of the genotypes of two hypervariable genes, UL146 and RL13 [93]. A genotype was assigned when the number of reads was >10% and >2% of the total number of reads detected for all genotypes of that gene, and the number of HCMV strains present in a clinical sample was determined as the number of genotypes detected for the two target genes. The motif read-matching was based on the conservation of genotype-specific motifs (20–31 nt), as identified by visual inspection of the alignments for 12 hypervariable genes (RL5A, RL6, RL12, RL13, UL1, UL9, UL11, UL73, UL74, UL120, UL146, and UL139), and the total number of HCMV strains identified in a clinical sample was scored as the number of genotypes detected for at least two genes.

The same study showed that recombination between two strains might occur in multiple-strain infections, with two-thirds of the genome remaining almost identical, and only one-third being different. On the other hand, the critical role of recombination during HCMV evolution was confirmed, and some recombinant strains appeared to have survived for thousands of years without further recombination, whereas other strains appeared to not have recombined for a long time, following their origin from a common ancestor. In addition, the recombination rate for some hypervariable genes was low, such as for genes coding for gB (3.3%), gN, and gO (2.9%), whereas, no recombinants were identified for UL146. Furthermore, pseudogenes, i.e., gene loss deriving from mutations causing premature translational termination in at least one gene, were reported in 77% of the strains sequenced from clinical material [92], with a frequency similar to that previously found for strains passaged in cell cultures [93].

Very recently, the genome-wide intra-host diversity, which was reported to approximate that of RNA viruses and was attributed to new mutations [88], was shown to be due to superinfections with new virus strains and subsequent within-host recombinations [94]. Through longitudinal sampling of chronically HCMV-infected immuncompromised children and reconstruction of single viral haplotypes, it was hypothesized that HCMV genome diversity was due to the multiple-strain presence in a single sample. First, to verify this hypothesis, a phylogenetic tree was constructed, which showed that in sequential samples from a single patient two strains were present, clustering into separate clades. Then, to confirm this result, (i.e., the presence in a single sample of mixed strains), whole-genome HCMV haplotypes from longitudinal sequence data were reconstructed, showing that for some patients, the number of haplotypes was two or three. In addition, haplotype sequences clustered into clearly distinct clades. Thus, it was concluded that the HCMV within-host diversity was similar to that of other DNA viruses and considerably less than that of RNA viruses, including HIV, while a high HCMV diversity over a brief evolutionary time-scale was not evidenced, whereas mixed infections were shown to be the basis for within-patient diversity [94]. In other words, it was shown that diversity within the same host was not due to evolutionary events occurring on a short-time scale, but due to multiple virus infections followed by recombination events. Thus, diversity appeared to be a marker of virus re-infection.

## 7. New Approaches to Diagnosis of HCMV NPI during Pregnancy

In order to diagnose HCMV NPI with certainty, in a pregnant HCMV-seropositive woman, the following study should be undertaken. A group of HCMV-seronegative women in child-bearing age and planning for more than one pregnancy in the near future should be enrolled and followed-up for some years, with serological controls (every 2–3 mos) aimed at detecting an HCMV seroconversion (PI) either in the presence or absence of clinical symptoms. Serological control should evidence the shift from HCMV-seronegativity to HCMV-seropositivity (appearance of HCMV-specific IgG antibodies, presence of HCMV-specific IgM antibodies along with an avidity index <25%). Serological diagnosis of PI should be accompanied by viral DNA detection through PCR in multiple clinical samples (blood, urine, saliva, cervical secretions) and possibly (but not necessarily) through virus isolation in human fetal fibroblasts from one or (preferably) multiple clinical samples from different body sites. While this diagnostic approach would be determinant for a correct diagnosis of HCMV PI (and cCMV from PI), it would be even more important for the genome characterization of the infecting virus strain in its multiple body localizations, in view of its potential subsequent genomic (differential) comparison with an HCMV strain responsible for an NPI in the same woman during a second pregnancy, with potential virus transmission to the fetus (cCMV from NPI). Thus, HCMV genomes from PI and NPI of the same pregnant woman as well as neonatal HCMV genomes of the relevant congenitally infected infants from maternal PI and NPI, could be compared, in order to verify the identity of maternal and neonatal strains as a consequence of either PI or NPI. The latter could be due to either re-infection with a new virus strain or reactivation of the strain originally responsible for the PI. Thus, the identity of maternal and neonatal strains with the original strain might not be only the result of a PI, but could also be the result of the reactivation of a primarily infecting virus that could have been transmitted to the offspring during pregnancy. However, to our knowledge, reports documenting this possibility are very few (28) at the moment.

Other important questions of the HCMV maternal–fetal transmission, which could be clarified by this study would be: (i) which genome mutations occur within the virus during the transmission from mother to fetus both in PI and NPI?; (ii) if both PI and NPI occur as mixed infections—in such a case, do recombination events occur?; and (iii) generally, are the mutations observed expressions of HCMV evolutionary events or are they the result of a recombination process?

In addition to the genome characterization through high-throughput sequencing methodologies, a parallel line of research should be undertaken in view of developing a new serological approach to the diagnosis of NPI. As mentioned above, thus far, the only serological procedure applied to the diagnosis of HCMV NPI was reported by Novak et al. [41], and was based on the ELISA detection of strain-specific antibodies directed to short hypervariable sequences of HCMV gB and gH. This method was reported as having the capacity to detect about 60% of NPI. However, it was employed to diagnose NPI in nearly all epidemiological studies of the last decade on the frequency of new HCMV infections in pregnant HCMV-seropositive women. In this respect, the development of a new serological method able to diagnose all cases of NPI in pregnant women would be highly desirable and much cheaper and easier to use daily in a diagnostic virology laboratory than through virus genome sequencing. 

## 8. Multiple Strain HCMV Infection and HCMV Vaccine

Like the other members of the *Herpesviridae* family, HCMV, following the robust immune response induced by PI, might not prevent either secondary virus infections by new HCMV strains (NPI) or reactivation of the latent virus already present in the body. This meant that both, the humoral and the T cell responses were inadequate to protect the individual against subsequent infections. It is still a commonly held opinion among immunologists and virologists that antibodies, and particularly NAb, are able to block the infectivity of a virus. This concept is true for most viruses, but not for HCMV (and the other herpesviruses). Why would that be so? The reason is that NAb might inactivate the virus when they meet cell-free enveloped virus particles, as occurs in vitro in the neutralization assay, where virus and antibodies (serum) are incubated in a mixture. Therefore, this is an artificial condition occurring mostly in vitro, but infrequently occurring in vivo, where HCMV is strictly cell-associated, and mostly spreads from cell-to-cell. This has been documented for HIV [95] but also for HCMV, showing that NAb might limit cell-to-cell spread only if present at very high concentrations [96,97]. This resistance to NAb was dependent on the high level of expression of PC [97]. This conclusion somewhat matches the results of our studies, which evidenced an inverse correlation of high NAb titer and intrauterine transmission and showed that neutralizing human mAbs directed to the pUL128L of PC were active in protecting against vertical transmission at a picomolar range, whereas human mAbs to gB, gH, or gO were active only in a nanomolar range [17,18,98]. This mAb activity included neutralizing as well as PFI, LTI, and SFI antibody activities. 

Furthermore, a protective role of HCMV-specific antibodies was reported in animal models [99,100] as well as in transplant recipients [101]. In the guinea pig model, which represents the only small animal model available for quantitative measures of virus transmission and efficacy of vaccination, both MVA-vectored PC and gB vaccines improved pregnancy outcome after a guinea pig CMV challenge, but only gB reduced vertical transmission [102]. On the other hand, inclusion of the guinea pig PC in a vaccine design greatly improved protection against cCMV in the guinea pig model [103]. In Rhesus monkeys, following CD4^+^ T-cell depletion, administration of hyperimmune IgG to pregnant dams completely protected against fetal loss and placental transmission [104]. Recently, in a mouse model it was reported that strain-specific antibody therapy prevented HCMV reactivation after transplantation, provided that serotherapy was matched to the infecting HCMV strain [105]. On the other hand, the administration of commercial preparations of polyclonal human IgG did not significantly prevent cCMV in pregnant women with PI [2], while gB antibodies following gB vaccination of transplant recipients conferred some protection, independent of NAb activity [106]. 

In summary, the NAb activity is not sufficient for supporting protective immunity both after natural infection or after vaccination [107]. In recent years, reports have been published that have claimed that protection from infection might be conferred by extraneutralizing antibody functions, such as AD-cellular cytotoxicity (ADCC) [108], AD-cellular phagocytosis (ADCP), [109], AD-complement deposition (ADCD) [107], and AD-NK cell activation [7]. In transplanted patient sera, we repeatedly observed the presence of very high NAb titers as well as ELISA IgG antibody response to PC, in the absence of protection against HCMV infection [110]. These results were recently confirmed by an ADCC-assay, which showed that the protective effect elicited by the gB vaccine cannot be explained either by NAb or ADCC in PI of transplanted patients [111]. On the other hand, it was shown that antibodies-induced by the gB vaccine mediated virion phagocytosis (ADVP), whereas they were poor mediators of NK cell activation (ADCC) [112].

The major role of the T cell response in the protection against both cCMV and HCMV infections in the immunocompromised host has been reported in several studies. Concerning cCMV, as mentioned above, several studies have shown that (i) an early maternal lymphoproliferative response (LPR) was associated in pregnant women with lack of virus transmission to the fetus [19]; and (ii) a delayed CD4^+^, but not CD8^+^ T cell response was associated in pregnant women with cCMV [20], as also confirmed more recently in a distinct pregnancy cohort [24]. These studies support a major role of CD4^+^ T cell response in both eliminating virus-infected cells and supporting the B cell response. As a general conclusion, what seems important in the prevention of cCMV is the rapidity of the T cell response to the virus replication rate, which appears to be the most critical parameter that is able to prevent placental infection [18,104].

Similar to the pregnant women population, the HCMV-specific CD4^+^ and CD8^+^ T cell responses were also repeatedly investigated in both solid organ transplant (SOT) and hematopoietic stem cell transplant (HSCT) recipients [110,113,114,115], confirming the primary role of the CD4^+^ T cell response in association with the complementary and supporting role of CD8^+^ T cells. A major breakthrough in the study of the mechanism of protection against HCMV infection might have been brought about by the development of antibody-dependent cellular assays, in which a cooperation of cellular and humoral arms of the immune response appears to be a potential major tool of defense against HCMV infections. In conclusion, both innate (NK and also γ/δ T cells) and adaptive T-cell responses, as well as antibody response, appear to contribute substantially to defense and protection against HCMV infections. However, while the mechanisms of defense against HCMV have been extensively investigated in PI, the distinctive characteristics of the immune response to NPI remains to be explored thoroughly, mainly in view of defining its immune correlates of protection. While the immune correlates of protection against PI have been partially identified (as reported above) but shown not to be protective against new secondary NPI, some new parameters of protection against NPI should be identified as early as possible. Hopefully, when identified, they would represent the primary objectives to be reached by a vaccine to be able to protect from both PI and NPI. 

Until this important result was achieved, a vaccine eliciting an immune response somewhat similar to that produced by natural PI should be sponsored and approved by health authorities, as repeatedly reported by some researchers working in the field for many years [38,116], while other groups of researchers as well as manufacturers have been discouraged by the limited protection conferred by natural infection, and the vaccines that have so far been tested against NPI [45,117,118]. This controversy was reported as a paradox or as the enigma of non-protective maternal immunity [119,120].

Thus far, live virus vaccines, such as AD169, Towne, and Towne/Toledo chimera vaccines have not given satisfactory results, either in clinical trials or in experimental animal models [121]. As for non-living HCMV vaccines, a theoretically optimal recombinant HCMV vaccine composition should include—(i) gB, inducing both humoral and T-cell immune responses [106,122,123]; (ii) PC, inducing the highest NAb response [16]; and (iii) pp65, inducing the most potent T-cell response [24,124,125]. At the moment, there are three vaccine candidates that include all of these three HCMV antigens—(i) V160; (ii) a purified dense body (DB) vaccine; and (iii) a modified vaccinia virus Ankara (MVA) vaccine [126].

The V160 vaccine was derived from the original AD169 strain [127], which showed a 1-bp insertion (A) inducing a frameshift mutation in UL131 [73]. In 2012, PC of the original virus strain was restored by Fu et al. [128] by a series of passages in endothelial cells, as previously indicated by our group [129]. The revertant AD169 virus recovered its tropism for leukocytes and endothelial cells, while eliciting higher NAb titers (compared to the mutated strain) and showing multiple native neutralization epitopes [130] as previously reported in humans, following natural infections [15]. Subsequently, V160 was created by repairing the frameshift in UL131 in a BAC-cloned derivative of AD169. The revertant AD169 with its restored PC was then genetically modified by inserting a chemically controlled protein stabilization switch, which allows virus replication in the presence of a synthetic compound (Shield-1), but stops virus replication in its absence. This replication-defective HCMV vaccine was named V160 and was shown to be immunogenic in several animal species [16,131]. In a Phase I study in seronegative subjects, the V160 vaccine was safe, well tolerated, and induced NAb as well as T-cell responses within the different ranges of natural immunity [132]. In April 2018, a double-blind, randomized, placebo-controlled Phase 2b, multi-center clinical trial was started under the sponsorship of Merck SD Corporation to evaluate the safety, tolerability, efficacy, and immunogenicity of a 2-dose and 3-dose regimen of V160 vaccine in healthy seronegative women 16–35 years of age. Completion of the study is expected in May 2021.

As for the DB vaccine, it consists of non-infectious enveloped particles (bodies) that accumulate within the cytoplasm of infected cells (namely human fibroblasts) during virus replication, and contains viral glycoproteins and tegument proteins but not viral DNA. Thus, DB were investigated [133,134] and proposed as a potential HCMV vaccine candidate. Using preparations derived from the laboratory strain Towne, DB were shown to induce both NAb as well as cell-mediated immune responses in mice [124,135]. However, since Towne-derived DB do not contain functional PC, due to the presence of a single-nucleotide mutation in the UL130 ORF of the UL128L, recently the UL130 mutation was repaired by using BAC mutagenesis and, thus, obtaining the Towne-UL130rep. This strain synthesized PC-positive DB in infected fibroblasts, which elicited an NAb response higher than that induced by PC-negative DB in both mice and rabbits. The same result was obtained in fibroblast, epithelial, and endothelial cells [136]. On this basis, a GMP-compliant protocol for DB production for use as a vaccine was proposed [137]. This protocol included—(i) deletion of the UL25 ORF to attenuate the virus; (ii) conditional expression of pUL51 by using the Shield-1/FKBP destabilization system to block the capacity of the virus to replicate in vivo; and (iii) use of the viral terminase inhibitor letermovir to reduce the contamination of the DB vaccine by infectious virus. This vaccine could represent a favorable option for clinical studies in the near future. Recently, the Serum Institute of India has been involved in the production of a DB vaccine.

Finally, multiantigenic MVA vaccine vectors simultaneously expressing PC, gB, and pp65, when inoculated in mice, were shown to elicit potent NAb as well as polyfunctional T-cell responses [126].

## 9. HCMV Recombinant gB Subunit Vaccine

However, at this time, the vaccine showing the best results in terms of protection against HCMV infection in humans has been a recombinant gB vaccine, which remains a basic component of all vaccines currently being developed [138]. Based on the demonstration that gB (pUL55) was able to induce NAb, preventing infection of human fibroblasts [139], in the late 1980s Chiron developed a recombinant HCMV vaccine produced in Chinese hamster ovary (CHO) cells and mixed with an oil-in-water microfluidized emulsion adjuvant 59 (MF59), which showed a fair, but waning, NAb response, in both adults [140] and toddlers [141]. Subsequently, Novartis and then Sanofi Pasteur modified the Towne gB by removing the transmembrane domain and the furin cleavage and fusing the cytoplasm with the extracellular component of gB. This vaccine was shown to be safe and immunogenic in Phase I studies [142]. On this basis, three Phase II studies were conducted. The first was done in HCMV-seronegative women immunized in the immediate post-partum period, and showed an efficacy of 50% in the prevention of PI. In addition, in the same trial, HCMV-seropositive women were vaccinated and shown to induce boosted gB-specific immune responses (both humoral and CD4^+^ T-cell) [122], although in the presence of pre-existing immunity [143]. The second study was conducted in HCMV-seronegative adolescent girls, who showed a 43% reduction (although not significant) in the incidence of PI, compared to a control group [123]. Finally, a third study was performed by Griffiths et al. [106] in SOT recipients, and showed in seronegative patients with seropositive organ donors that the duration of HCMV viremia and antiviral treatment were significantly reduced in vaccinees compared to placebo recipients. More recently, Sanofi is working into two new directions by—(i) testing newer adjuvants aimed at enhancing both the NAb and the CD4^+^ T-cell responses; and (ii) combining a recombinant PC with gB in a novel vaccine formulation.

This beneficial effect of the gB vaccine was considered to be due primarily to the NAb response. We have already reported above how NAb per se might not be considered responsible for the protective effect of natural infection or HCMV vaccination, but some other mechanisms such as T cell and AD-cellular responses might play a major role. Similar conclusions were reported in two papers that recently appeared in the same journal [111,112], which showed that in both of the above mentioned studies [106,122] the IgG response was of the same magnitude as in natural infection, whereas the NAb response to gB was minimal (sera were examined retrospectively). Both studies also examined the ADCC response, which was previously found to be protective for the RV144 HIV vaccine [144], but in both studies ADCC was not demonstrated in seronegative vaccine recipients, but only in seropositive controls. An alternative non-neutralizing IgG-mediated function was, however, found in post-partum women, i.e., AD cellular phagocytosis (ADCP) [112]. 

Furthermore, both studies also examined the antibody response to gB antigenic domains, referred to as AD-1 to AD-5, which were identified as targets of NAb [145]. In the general population, while 100% of the individuals possess antibodies (not only neutralizing) to AD-1 [146], only about 50% have antibodies to AD-2 [147]. In a previous study, Baraniak et al. [148] reported that in HCMV-seropositive SOT recipients, the antibody response to AD-2 was significantly lower in patients developing HCMV disease, compared to viremia-free patients. However, in the PNAS study, Baraniak et al. [111] did not detect any antibody response to AD-2 in HCMV-seronegative patients, following vaccination. Thus, antibody response to AD-2 did not appear to be a reliable correlate of protection in seronegative SOT recipients. On the other hand, in the Nelson et al. study [112], conducted in sera from HCMV-seronegative women vaccinated post-partum during the Pass et al. study [122], a significantly higher antibody response to gB AD-3 compared to HCMV-seropositive controls was reported. However, since AD-3 is an intracellular domain of gB, which cannot be reached by antibodies, AD-3 antibodies cannot represent a correlate of protection by neutralization or HCMV binding, and some other mechanism of protection, such as ADCP, could play a role. In addition, a statistically significant increase in binding to linear peptides of the AD-2, site 2 (which is a non-neutralizing epitope) region, was reported in HCMV-seronegative women, during a follow-up. This finding seems to be in agreement with the protective AD-2 vaccine response reported in SOT recipients, as mentioned above [148]. 

Some operative conclusions drawn from the clinical trials completed thus far, in view of the development of future trends for the improvement of gB vaccines, are as follows [138]: (i) to test the gB/MF59 vaccine in clinical trials performed in both seropositive and seronegative healthy people, and not only in pregnant women and SOT recipients; thus far, a single trial in healthy seronegative adolescent girls has been performed; (ii) since the gB/MF59 vaccine is based on the Towne gB sequence, is the vaccine able to protect against multiple different HCMV strains, i.e., against both PI and NPI ?; (iii) since gB present in the vaccine is highly glycosylated, how can the gB epitopes covered by the glycan shielding be made available to the immune system? It is tempting to hypothesize that the pre-fusion (native) form of gB would make neutralization sites more exposed to NAb, compared to the post-fusion form [149,150]; and (iv) using a bioinformatic sequence analysis of 207 HCMV strains for evaluation at the aa level of the percentage of conservation of gB and PC, a mean identity of 96–98% was found, thus documenting that the genetic polymorphism was likely not to be responsible for the modest efficacy of gB vaccines in recent clinical trials, while it was suggested that gB deprived of AD-1 and AD-3 (see below) might elicit a higher NAb response [151]. 

The gB is a type I envelope glycoprotein and class III viral fusogen [152] and consists of a 906–907 aa polypeptide (according to different HCMV strains). Following extensive post-translational modifications, including several glycosylation steps at N- and O-linked sites, gB is cleaved by the cellular endoprotease furin into two moieties—the amino (gp90)- and the carboxy (gp58)-terminal subunits, which are then linked by disulfide bonds, thus, forming the mature glycosylated gB with a trimeric conformation. It then dimerizes in its final conformation within the viral envelope [153], interacting with gH/gL to form a stable complex, the core fusion machinery [154]. As mentioned above, gB used in the gB/MF59 recombinant vaccine was derived from the Towne strain, deleted of both the furin cleavage site and the trans-membrane region, thus, forming an 807 aa protein with 19 N-linked glycosilation sites. As for AD1–5, AD-1 includes aa 560–640; AD-2 contains two discontinuous binding sites, i.e., site I (aa 68–77) largely conserved among strains and target of NAb, and site II (aa 50–54) not conserved; AD-3 maps to an intraluminal part of gB (aa 708–805), thus, is not expected to be exposed to NAb; AD-4 is a discontinuous domain mapping to aa 121–132 and 344–438; finally, AD-5 is a structural domain mapping to aa 133–343.

HCMV gB has been shown to share structural and functional properties with gB of other herpesviruses like HSV-1 and EBV—on this basis, it has been proposed that HCMV gB, like gB of HSV-1 and EBV, has two internal hydrophobic fusion loops interacting with target membranes [153]. Thus, analogous to the gB of the other two herpesviruses, it was hypothesized to undergo large refolding events during fusion. What is important to retain in view of the development of an HCMV vaccine is that the recombinant gB ectodomain displays post-fusion conformation, which is very different from the pre-fusion conformation present on the viral envelope or on the infected cell surface [153]. Recently, structural and functional states of HCMV gB have been deeply investigated by cryo electron tomography [155]. By using this technology, two distinct gB trimer conformations were identified at 21 Å resolution—a predominant (79%) conformation (designated pre-fusion) resembling a globular endodomain and a Christmas tree-shaped ectodomain, and a minor conformation (21%) resembling a columnar tree-shaped ectodomain matching the crystal structure of the post-fusion gB ectodomain. Studies of the humoral immune correlates of protection against HCMV primary infection through a comparative evaluation of the antibody binding to pre-fusion gB expressed on a cell surface and to the vaccine soluble gB antigen (post-fusion) are ongoing. Another step forward towards the development of a new vaccine formulation consisting of a trimeric (pre-fusion) gB conformation was made by producing a new trimeric recombinant gB protein in CHO cells, in which the coding sequence for the cleavage site was replaced with a 15 aa linker sequence. Mice immunization with this gB elicited a gB-specific IgG antibody titer higher than monomeric gB, as well as a higher NAb titer in both fibroblasts and ARPE-19 epithelial cells. This NAb activity was also confirmed against other clinical HCMV isolates [156].

In conclusion, future trends for the development of a new gB recombinant vaccine containing gB in the pre-fusion form should be directed to verifying whether—(i) nearly the totality of PI are prevented in sero-negative individuals; and (ii) NPI are also prevented in HCMV-seropositive individuals.

At the moment, other vaccines have been or are being considered for transplant recipients and particularly for seropositive HSCT recipients—ASP0113, Pepvax, and Triplex. ASP0113 is a DNA vaccine expressing two proteins, gB and pp65, which was aimed at boosting the pre-existing immunity in HSCT recipients through therapeutic vaccination. Following Phase I studies showing safety, it was tested in Phase II and Phase III studies. However, the results did not show any significant improvement in vaccinees in both overall survival or reduction in HCMV end-organ disease, compared to controls. The failure in protection of ASP0113 was attributed to the poor immunogenicity of DNA vaccines [157]. Pepvax is a chimeric peptide vaccine consisting of a cytotoxic HLA-restricted T-cell epitope from pp65 (HLA A* 0201 pp65_495-503_) fused with the P2 peptide T-helper epitope of tetanus toxin. Encouraging results were obtained in Phase I studies, which showed a reduced incidence of HCMV reactivation episodes and usage of antivirals, along with a rapid HCMV-specific T-cell reconstitution [158]. Finally, the Triplex vaccine consisted of a modified MVA encoding three full-length proteins—pp65, IE1-exon4, and IE2-exon5. Following encouraging results in Phase I in terms of safety and immunogenicity, a Phase 2 randomized clinical trial was undertaken in HCMV-seropositive HSCT recipients from matched related/unrelated donors in the absence of T-cell depletion. Patients received Triplex or placebo on day 28 or 56 after HSCT and were followed up for one year. The trial was completed in the fall of 2019, and showed that the Triplex vaccine amplified HCMV-specific immune responses, while fewer HSCT recipients had HCMV viremia compared to placebo-treated patients [159]. Based on these results, the City of Hope has initiated a clinical trial (NCT03560752), in which donors of HCMV-positive HSCT recipients will receive one injection of Triplex, in view of developing an HCMV-specific T-cell response that will be transferred to the recipient, in order to prevent an early viremia, prior to initiating antiviral prophylaxis with letermovir. It is planning to adopt a similar approach in pediatric HSCT recipients.

## 10. Pathogenesis of cCMV Infection at the Uterine-placental Interface

**Figure 4:**
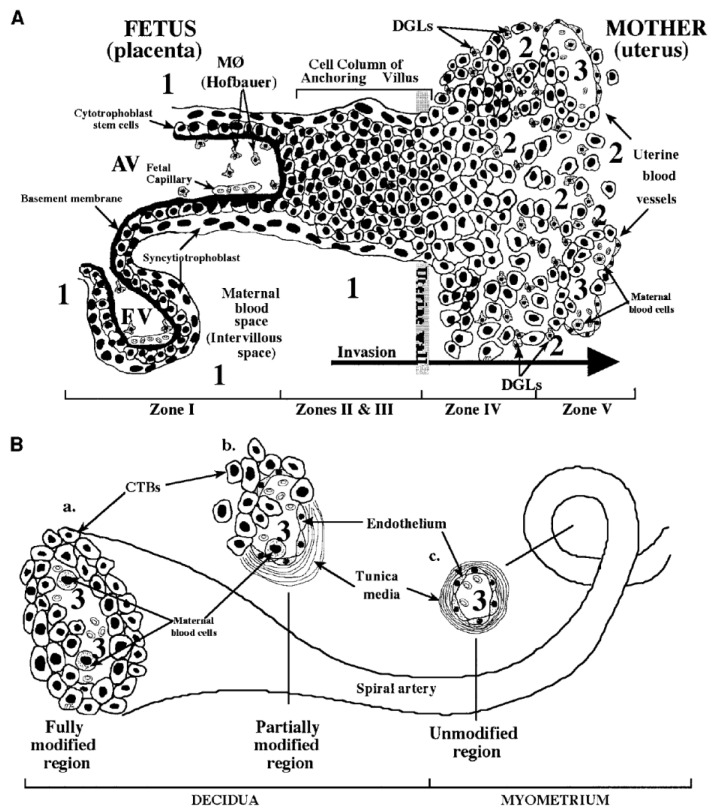
Fetal–maternal interface (FMI) at the end of the first trimester of human pregnancy. (**A**) Diagram of the FMI showing—(i) a floating villus (FV) which contains macrophages (Hofbauer cells) and fetal blood vessels and is bathed by maternal blood; and (ii) an anchoring villus (AV) resembling a bridge between the fetal and maternal compartments and consisting (zone I) of cytotrophoblasts (CTB) that form cell columns that attach to the uterine wall (zones II and III). CTB invade (zone IV) the uterine wall (decidua and myometrium), thus, accessing the maternal circulation (zone V). Areas of potential HCMV transmission to the placenta are numbered as—1 (direct transmission from maternal blood to CTB); 2 (transmission from macrophages (MØ) and decidual granulocytes (DGLs) to invading CTB); and 3 (transmission from maternal blood cells to fetal endothelial cells and CTB). (**B**) Diagram of a uterine spiral artery, in which endovascular CTB invasion is in progress—(a) a fully modified region; (b) a partially modified region in the decidua as a result of CTB invasiveness; and (c) an unmodified region of the spiral vessel in the myometrium are shown [163].

The pathogenesis of cCMV infection is still far from being entirely resolved. The basic structure of the placenta consists of several chorionic villi, each containing a stromal core with blood vessels delimited by a basal membrane surrounding two cell layers—cytotrofoblast (CTB) progenitor cells and syncytiotrofoblasts (STB). Floating villi (FV) are in direct contact with maternal blood, while anchoring villi (AV) maintain the fetus attached to the uterus by means of cell columns bringing maternal blood to the intervillous area (Figure 4). CTB might fuse to the original STB surrounding the FV or form cell columns in AV. STB are the site of maternal–fetal exchanges, while CTB invade the uterine wall and breach uterine arterioles by replacing the original smooth muscular and endothelial cells with new endothelial cells, thus, directing maternal blood towards the placenta [160]. This process is a sort of vasculogenesis and must occur in the absence of maternal immune response. 

The analysis of samples from the uterine-placental interface has documented that HCMV first infects decidua and replicates in glandular epithelial cells, endothelial cells, and intravascular CTB [160,161,162]. HCMV then spreads to the invasive CTB and impairs invasion by decreasing the expression of integrins [161] and other paracrine factors required for invasiveness, thus, impairing the placental and fetal development. 

It is still a matter of debate whether maternal humoral immunity, and particularly IgG NAb, might provide protection against cCMV. It has been proposed that HCMV forms complexes with IgG, which are captured by the neonatal Fc and transcytosed within STB, where the complex might be degraded and the virus inactivated, or transcytosed into CTB, where the virus might replicate and spread to stromal fibroblasts and fetal capillaries in the villous core [161,162,163,164]. In 2005, it was reported that administration of HIG to pregnant women with HCMV PI reduced virus transmission to the fetus and prevented cCMV [11]. Placentas with cCMV showed some increase in weight and thickness [165] associated with a hypoxia-like condition [166], which resulted in a compensatory placental enlargement. These pathological findings appeared to be reduced following HIG treatment. However, these results were not confirmed by a randomized trial of HIG to prevent cCMV, which showed no significant difference in the incidence of cCMV as well as in the histology of term placentas between HIG-treated and -untreated groups of pregnant women [2,167]. More recently, a new, but uncontrolled study (treated vs. historical groups), reported that the transmission rate in the HIG-treated group (7.5%), was significantly lower than that of the historical control group (35.2%) [168]. This better result was attributed to the administration of a higher dose of HIG (200 IU) every other week, compared to a dose of 100 IU every month, given by Revello et al. [2]. A future controlled study using this dosage is warranted. 

Ex vivo human placenta explants, and particularly sections of decidua (maternal compartment) were cultured to study HCMV infection and spread, showing that decidual fibroblasts, epithelial, and endothelial cells, as well as CTB, supported virus replication [169]. In addition, HCMV has been shown to replicate in vivo in xenografts of human placental villi transplanted under the kidney capsule of SCID mice [170]. In this in vitro model, CTB induced remodeling of blood vessels (neoangiogenesis), and also formation of lymphatic vessels (neolymphangiogenesis) similar to those seen in human decidua. VR1814 infection of xenografts markedly reduced invasiveness and remodeling of CTB, whereas AD169 infection substantially failed to do so. The in vitro model of ex vivo explants could be useful in the near future for evaluating the protective effect of antibodies against placental infection. As for the fetal compartment, placental villous explants were infected in parallel with the attenuated laboratory strain AD169 and the low-passage clinical strain VR1814 (from our laboratory). Comparative results showed that many VR1814-infected CTB expressed both IE1 and IE2 as well as gB, whereas only a few AD169-infected cells expressed IE1 and IE2 HCMV antigens [171]. In addition, while AD169-infected explants were comparable to controls, explants infected with VR1814 showed significantly smaller villi associated with infection of column CTB, thus, impairing the development of anchoring villi. To counteract this effect, HIG [166] and potently neutralizing human mAbs [172] were shown to rescue the development of infected anchoring villi. Very recently, neutralizing human mAbs to gB, gH/gL, and PC have been reported to reduce HCMV infection and spread in primary placental cells and explants of developing anchoring villi, showing that the protective effect of mAbs to PC was greater than that of mAbs to gB and gH/gL [171]. As for the local cellular immunity at the uterine–placental interface, in VR1814-infected intact explants of basal decidua from seropositive women, at third day post-infection, HCMV was found to replicate in decidual stromal cells and epithelial cells of endometrial glands, which were in close contact with CD8^+^ T-cells and, to a lesser extent, NK cells. These results seemed to indicate an early T-cell response to virus infection, and are hypothetically transferrable to the in vivo placental infection in NPI, when T-cell immunity is well developed [173]. 

## 11. Conclusions

As reported above, knowledge already present at the end of the 1990s, that HCMV PI did not elicit protective immunity against subsequent virus contacts, prompted some researchers and manufacturers to abandon the idea that the availability of an HCMV vaccine was an urgent need to satisfy. This opinion was based on the consideration that protection from PI only, in the absence of protective immunity against NPI, did not justify the burden of efforts required for the development of an HCMV vaccine of limited efficacy [118]. Conversely, other researchers, based primarily on the partial protection given by natural HCMV PI and the partial identification of some correlates of protection from PI, share the point of view that even a partial protection of pregnant seronegative women from PI is sufficient to justify the scientific and financial efforts aimed at the development of an HCMV vaccine [37]. Results of 50% protection from PI of seronegative pregnant women receiving the gB/MF59 vaccine, as shown in the above reported clinical trial [122], appear to be a good step forward towards prevention of cCMV. According to this opinion, any case of prevented cCMV would be a major success for Public Health. Obviously, vaccines preventing PI and also boosting the immune response of seropositive individuals would be highly desirable [174]. Major targets of such a vaccine would be young women and adolescents to prevent cCMV, as well as toddlers, to prevent transmission to adults and, thus, decrease virus dissemination.

## List of Abbreviations:

cCMV	congenital Cytomegalovirus
HCMV	human cytomegalovirus
PI	primary infection
T	transmitting women
NAb	neutralizing antibodies
NT	non-transmitting women
HIG	human immunoglobulin
gCs	glycoprotein complexes
PC	pentamer complex
mAbs	monoclonal antibodies
IMAB	inhibition of monoclonal antibody binding
PFI	plaque formation inhibition
LTI	leukocyte transfer inhibition
SFI	syncytium formation inhibition
ELISPOT assay	enzyme-linked immunospot assay
CID	cytomegalic inclusion disease
SNHL	sensoryneural hearing loss
TC	trimer complex
HPC	hemopoietic progenitor cells
EBV	Epstein-Barr virus
IE	immediate-early
UL	unique long region
BAC	bacterial artificial chromosome
US	unique short region
SNP	single nucleotide polymorphism
ADCC	antibody-dependent cellular cytotoxicity
ADCP	antibody-dependent cellular phagocytosis;
ADCD	antibody-dependent complement deposition
LPR	lymphoproliferative response
DB	dense bodies
MVA	modified vaccinia virus Ankara
SOT	solid-organ transplant
HSCT	hematopoietic stem cell transplant
CTB	cytotrophoblasts
STB	syncytiotrophoblasts
FV	floating villi
AV	anchoring villi
